# The effect of methylphenidate on pain perception thresholds in children with attention deficit hyperactivity disorder

**DOI:** 10.1186/s13034-023-00667-y

**Published:** 2023-10-13

**Authors:** Abdullah Bozkurt, Selin Balta

**Affiliations:** 1 Department of Child and Adolescent Psychiatry, Ataturk University, Erzurum, Turkey; 2 Department of Pain Medicine, University of Health Sciences, Konya, Turkey

**Keywords:** Pain perception threshold, Attention deficit hyperactivity disorder, Methylphenidate

## Abstract

**Background:**

Pain perception is important in children with attention deficit hyperactivity disorder (ADHD) since they are more likely to experience painful events due to increased accident rates. The aim of this study is to contribute to the literature concerning the relationship between ADHD diagnosis, methylphenidate (MPH) therapy, and pain thresholds, since findings regarding the change in pain perception in children with ADHD are scarce and inconsistent.

**Methods:**

Children aged 8–13 years constituted both the ADHD group (n = 82) and the healthy controls (n = 41). The ADHD group was divided into two subgroups, ADHD without MPH (not treated pharmacologically) and ADHD with MPH (treated pharmacologically for at least three-months). The Conners’ Parent Rating Scale–Revised: Short Form was employed to assess ADHD, a visual analog scale was applied to evaluate chronic pain severity, and a manual pressure algometer was used to assess pain thresholds.

**Result:**

Children with ADHD had lower pain thresholds than the healthy controls (*P < 0.05)*. However, lower regional pain thresholds were observed in the ADHD group without MPH compared to both the healthy control and ADHD with MPH groups. Although pain thresholds in the ADHD with MPH group were regionally lower than in the healthy controls, low pain thresholds were found in fewer regions compared to the ADHD without MPH group.

**Conclusions:**

Children with ADHD are more sensitive to pain sensation, and MPH may help normalize these individuals’ pain experiences by raising pain thresholds. Families and clinicians must be aware of situations that may cause pain in children with ADHD. In addition, these children’s low threshold for pain may lead them to experience it more intensely.

## Introduction

Attention deficit hyperactivity disorder (ADHD) in pediatric cases is characterized by hyperactivity, impulsivity, and/or inattention and affects 5% of children worldwide. The symptoms impact on cognitive, educational, behavioral, and emotional functioning [1]. Children with ADHD also exhibit problems with motor coordination, balance, and somatosensory functions outside the primary clinical area [2–4].

Despite being one of the most extensively researched psychiatric disorders, the mechanism underlying the symptoms of ADHD is not completely understood [5]. Numerous studies have shown that ADHD has substantial biological origins, including problems in neurotransmitter systems in the brain. Genetic, imaging, and pharmacological studies support the idea that ADHD is primarily caused by dopamine dysfunction [6]. Moreover, a functional dopaminergic neurotransmitter system is of crucial importance, not solely for higher cognitive functions, but also for components of human perception such as olfaction, audition, vision, and sensory sensitivity [7–10]. Given the complex character of ADHD, the majority of current research has focused on cognitive and behavioral abilities linked to attentive and executive functions, while the sensory dimension has received little attention. Increasing evidence indicates that dopamine also plays a role in other perceptive deficiencies, such as color perception, tactile perception, and pain perception, in addition to the fundamental symptoms of ADHD [11–13].

Stimulants used to treat ADHD, such as methylphenidate (MPH) and amphetamine, help improve attention, concentration, and impulse control by increasing levels of neurotransmitters such as dopamine and norepinephrine in the brain [14]. Methylphenidate has been shown to exhibit positive effects on emotional processing, cognitive motivation, working memory, and social behavior, in addition to core symptoms in ADHD [15–18]. The therapeutic effects of MPH are attributed to its ability to produce slow, steady increases in dopamine that mimic tonic firing in the brain [19]. However, the side-effects of MPH, such as appetite suppression, insomnia and negative emotional symptoms, should also be remembered [20].

Accumulating data suggest that dopamine is also implicated in pain processing [21, 22]. Many regions of the central nervous system involved in pain processing contain a high density of dopamine receptors, whose activation can be analgesic in both humans and animals [23, 24]. Moreover, a small number of clinical researchers have asserted that dopaminergic medications help alleviate chronic pain [25, 26].

The dopaminergic anomalies exhibited by individuals with ADHD are likely to result in altered pain perception. Pain sensitivity in children with ADHD is one of the least frequently studied aspects of the condition. However, this may be particularly important to these children, as they are more likely to experience painful events due to their higher accident rates [27].

A limited number of studies have examined pain perception in children with ADHD. Scherder et al. evaluated the pain perception of children and adolescents with ADHD, their unaffected siblings, and healthy controls using the children’s pain inventory [28]. Those authors found no differences in pain perception between the groups. In the following part of that study, children and adolescents with ADHD and their unaffected siblings were asked to donate blood and to evaluate the intensity and emotionality of pain experienced following venipuncture. Children and adolescents with ADHD reported lower pain perception compared to unaffected siblings [28]. Northover et al. compared sensory pain between adolescents with pure ADHD and those with ADHD plus conduct disorder (CD) through the application of thermal heat to the skin of the palm of the hand. Although adolescents with ADHD plus CD exhibited considerably longer pain threshold times and were more tolerant to pain than those with pure ADHD, the physiological responses and skin conductance levels were identical in the two groups [29].

Research has also investigated pain in adults with ADHD and revealed a decrease in pain thresholds. In one study, in which pain sensitivity was evaluated psychophysically, the ADHD participants without methylphenidate (MPH) had a considerably lower cold pain threshold and cold tolerance compared to controls. Both the cold threshold and tolerance of ADHD patients treated with MPH increased significantly in comparison to those who did not receive medication. That study suggests that individuals with ADHD are more sensitive to pain and that MPH may have antinociceptive qualities [12]. Recent research indicates that ADHD increases pain perception and that generalized pain is more common in patients with ADHD than in control individuals [30, 31].

The purpose of this study is to contribute to the literature concerning the relationship between ADHD diagnosis, MPH therapy, and pain thresholds, since findings regarding the change in pain perception in children with ADHD are both scarce and inconsistent. In terms of deficits in dopaminergic pathways in ADHD and the mechanism of action of MPH in these pathways, we hypothesized that the pain threshold would be lower in patients with ADHD and would differ between the MPH, non-MPH, and healthy control groups.

## Methods

### Study design, setting, and ethics

This cross-sectional study was conducted in accordance with the Declaration of Helsinki and in line with the Strengthening the Reporting of Cross-Sectional Studies (STROBE) statement. The research was conducted between December 30, 2020 and March 30, 2021 at the University of Health Sciences, Konya City Hospital, Türkiye. Ethical approval was obtained from the Necmettin Erbakan University Ethics Committee (no. 14567952-050/1725).

### Participants

The participants consisted of children aged 8–13. The ADHD group was selected from individuals with ADHD symptoms who presented to the child and adolescent psychiatry clinic, while the healthy control group was established from individuals presenting to the general pediatric outpatient clinic. Children with ADHD were identified on the basis of clinical assessments using the *Diagnostic and Statistical Manual of Mental Disorders-5* (DSM-5) [32]. Diagnoses of ADHD and psychiatric disorders in the groups were assessed using the Schedule for Affective Disorders and Schizophrenia for School-Age Children-Present and Lifetime Version (K-SADS-PL) [33]. Members of the ADHD group with comorbid psychiatric, endocrinological, neurological, rheumatological, or oncologic diseases, and patients using medication on a continuous basis were excluded from the study.

The ADHD group was further divided into ADHD without MPH (not treated pharmacologically) and ADHD with MPH (treated pharmacologically for at least three month) subgroups. The healthy control group consisted of children admitted to the general pediatric outpatient clinic who participated in the study on a voluntary basis. These had no psychiatric, endocrinological, neurological, rheumatological, or oncological diseases and were not using medication.

The study sample size was calculated via G*Power - Universität Düsseldorf software. In a previous study [34] the response within each subject group was normally distributed with a standard deviation of 1.85. The true difference in pain pressure thresholds between the experimental and control mean values was 1.5, meaning that 41 experimental subjects and 41 control subjects would be required to be able to reject, with a probability (power) of 0.95, the null hypothesis that the population means of the experimental and control groups were equal. The Type I error probability associated with this null hypothesis test is 0.05.

### Measures

#### ADHD symptoms

The Conners’ Parent Rating Scale–Revised: Short Form (CPRS-R:S) is frequently used to investigate ADHD symptoms in children. On this four-point Likert-type scale ranging from 0 (not at all true) to 3 (very true), the parent is asked to assess how much each of the 27 items has represented a problem for their child [35]. The Turkish-language version of the CPRS-R:S has been confirmed as valid and reliable for the Turkish population [36].

#### Chronic pain severity

A visual analog scale (VAS) (0–100 mm) was used to examine the severity of pain, with 0 denoting “no pain” and 100 “the most severe pain possible.” The VAS is recognized as an easily applied and reliable scale for assessing the severity of pain in children aged ≥ 8 years [37]. A 30-second break was provided between trials to reduce sensitization due to repeated testing.

#### Pain threshold measurement

**Fig. 1:**
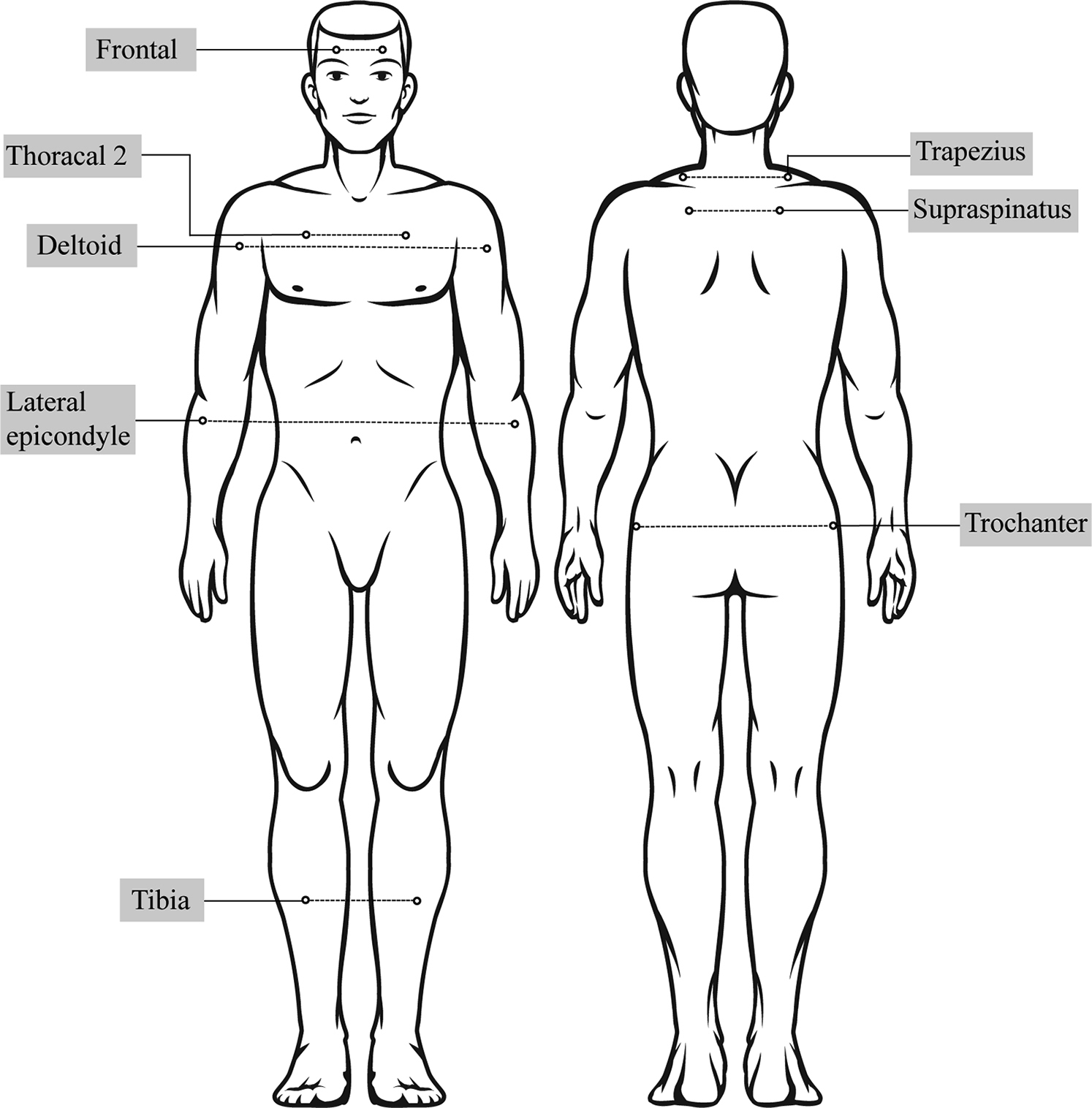
PPTs measured from eight reference regions

The pressure pain threshold (PPT) method using a manual pressure algometer (Baseline Dolorimeter®) was employed to assess pain thresholds. The investigator placed the algometer on the site to be examined and pressed against that site in a vertical direction while increasing the force at a constant rate of 1 kg/cm^2^. Algometry has previously been shown to be reliable in measuring PPTs in children [38]. The participants were instructed to express pain either by saying ‘stop’ or by raising their hands when they felt slight discomfort. The algologist gradually increased the pressure by 1 kg/cm^2^/s until the participants felt the first sensation of pain (PPT). A maximum pressure of 22 kg/cm^2^ was applied for all the participants. PPTs were measured bilaterally from eight reference areas using the algometer, the mid-frontal line, supraspinatus origin, trapezius middle fiber midpoint, and deltoid midpoint, 2 cm distal to the lateral epicondyle, the midclavicular line intersected with thoracal 2, the great trochanter region, and the proximal medial tibial point (Fig. 1). The value for each reference area was obtained by calculating the arithmetic mean of the bilateral measurements.

### Procedure

Children and their parents provided written consent to participation in the study. After the participants’ diagnoses had been evaluated, comorbidities were assessed using K-SADS-PL, and the CPRS-R:S was applied. The clinician then asked about the presence of chronic pain (defined as persisting for more than three months), and the average pain intensity. The algologist, one of the two authors of this study, who was blinded to the study groups, measured PPTs using a manual algometer.

### Statistical analysis

Statistical analyses were performed using SPSS version 28.0 software (IBM Corp., Armonk, NY, USA). There were no missing data for any variable. The Shapiro–Wilk test was used to evaluate the distribution of the data. Descriptive data are presented as frequencies (n) and percentages (%) for categorical variables, as median values with 25–75% percentiles for non-normally distributed numerical variables, and as means plus standard deviation for normally distributed numerical variables. The chi-square test was used to compare categorical variables between independent groups, while the Mann–Whitney U test was used to compare linear variables between independent groups. Correlation between numerical variables was assessed using Spearman’s and Pearson’s correlation analysis. p < 0.05 was considered statistically significant. The statistical differences between all ADHD patients and healthy controls in terms of PPT values were analyzed with Student’s T test in the trochanter area and the Mann-Whitney U test elsewhere.

Three-group comparisons were analyzed using ANOVA for normally distributed numerical variables, and with the Kruskal Wallis test for non-normally distributed numerical variables. Bonferroni correction was applied to avoid possible type-1 errors, and p < 0.016 was considered statistically significant. At evaluation of normality before the comparison of the healthy control, ADHD with MPH, and ADHD without MPH groups, only PPT values in the trochanter region met the normality criteria, but these did not meet the homogeneity of variance. Post hoc analysis was therefore performed with the Game-Howell test, since the Welch value was 0.017. Three-group comparisons in other areas of measurement domains were analyzed with the Kruskal-Wallis test, and Bonferroni correction was applied for within-group comparisons.

The relationship between the CPRS:S score and PPT values in the frontal, trapezius, supraspinatus, deltoid, tibia, lateral epicondyle, and thoracal 2 regions in the patients with ADHD was analyzed by means of Spearman’s test, while values in the trochanter region were analyzed using Pearson’s test. In the ADHD with MPH group, the relationship between CPRS:S scores and PPT values in the thoracal 2 and deltoid areas were analyzed using Spearman’s test, while values in the frontal, trapezius, supraspinatus, lateral epicondyle, trochanter, and tibial areas were analyzed with Pearson’s test. In the ADHD without MPH group, the relationship between CPRS: S scores and all PPT values was analyzed using Spearman’s test.

## Results

One hundred twenty-three individuals were included in the study, with 41 age- and gender-matched participants in each group. The average age was 10 (8.0-12.25) years. Each group consisted of 11 (26.8%) girls and 30 (73.2%) boys. Mean CPRS-R:S scores were 42.11 ± 18.72 for all ADHD patients, 47.34 ± 18.38 in the ADHD without MPH group, and 36.87 ± 17.77 in the ADHD with MPH group.

Chronic pain was present in 34 (41.46%) of all ADHD patients, in 19 (46.34%) members of the ADHD without MPH group, 15 (36.58%) of the ADHD with MPH group, and 19 (46.34%) of the healthy control group. No statistically significant difference was observed between the three groups in terms of the presence of chronic pain (p = 0.743).

Pain severity was 50 (30.0–50.0) mm in both the ADHD without MPH and ADHD with MPH groups, compared to 40.0 (40.0–50.0) mm in the healthy control group. No statistically significant difference in pain severity was observed between the two ADHD groups (p = 0.907).

The analysis results of between all ADHD patients and the healthy controls in terms of PPT values are given in Table 1.

**Table 1:** PPT values of volunteers in ADHD and healthy control groups

Area	PPT (gr/cm^2^)		*p*
	ADHD	Healthy Controls	
Frontal	7.0 (5.0-10.12)	9.0 (7.0–11.0)	0.008
Supraspinatus	8.25 (5.87–11.62)	13.0 (10.5–15.0)	< 0.001
Trapezius	7.75 (5.0-11.5)	13.0 (10.0–15.0)	< 0.001
Thoracal 2	8.0 (5.0–11.0)	9.0 (8.0–12.0)	0.008
Deltoid	10.0 (7.0–14.0)	14.0 (10.5–17.0)	< 0.001
Lateral epikondyle	9.5 (7.0–13.0)	12.0 (10.0-14.5)	0.010
Trochanter	15.53 ± 4.47	17.63 ± 3.93	0.008
Tibia	16.0 (12.5-19.25)	18.0 (15.0–22.0)	0.016

Measurements and comparisons between the three groups’ PPT values are given in Table 2.

**Table 2:** Comparison of Pain Pressure Thresholds

Area	PPT (gr/cm^2)^			p*		
	ADHD without MPH	ADHD with MPH	Healthy Control	ADHD without MPH vs. ADHD with MPH	ADHD with MPH vs. Healthy Control	ADHD without MPH vs. Healthy Control
Frontal	6.0 (4.5-9.0)	8.5 (6.0-11.75)	9.0 (7.0–11.0)	**0.013**	0.115	**< 0.001**
Trapezius	6.0 (4.75–9.25)	9.5 (6.25–12.25)	13.0 (10.0–15.0)	0.020	**0.002**	**< 0.001**
Supraspinatus	7.0 (5.0-10.25)	10.0 (6.5-12.75)	13.0 (10.5–15.0)	0.077	**0.001**	**< 0.001**
Thoracal 2	6.0 (4.0-10.5)	8.5 (6.25-12.0)	9.0 (8.0–12.0)	0.025	0.657	**< 0.001**
Deltoid	8.5 (6.0–13.0)	12.0 (8.0-14.5)	14.0 (10.5–17.0)	0.078	**0.007**	**< 0.001**
Lateral epicondyle	8.0 (6.0-12.75)	11.0 (7.25-13.0)	12.0 (10.0-14.5)	0.086	0.030	**< 0.001**
Trochanter	13.81 ± 5.99	15.04 ± 4.30	16.97 ± 4.22	0.167	0.178	0.017
Tibia	16.5 (11.5–20.0)	16.0 (13.75-18.0)	18.0 (15.0–22.0)	0.090	0.037	0.036

*=A significant p-value was accepted as < 0.016 according to Bonferroni correction

No relationships were determined between CPRS:S scores and PPT values in the frontal, trapezius, supraspinatus, deltoid, trochanter, tibia, lateral epicondyle, or thoracal 2 regions in the ADHD patients (p = 0.739, 0.992, 0.969, 0.981, 0.075, 0.311, 0.921, and 0.812, respectively). No relationships were observed between CPRS:S scores and PPT values in the thoracal 2, deltoid, frontal, trapezius, supraspinatus, lateral epicondyle, trochanter, or tibia areas in the ADHD with MPH group (p = 0.700, 0.831, 0.745, 0.823, 0.827, 0.867, 0.250, and 0.532, respectively). Finally, no relationships were found between CPRS:S scores and PPT values in the frontal, trapezius, supraspinatus, thoracal 2, deltoid, lateral epicondyle, trochanter, or tibia areas in the ADHD without MPH group (p = 0.079, 0.365, 0.332, 0.808, 0.922, 0.395, 0.121, and 0.520, respectively).

## Discussion

This study compared the presence and perception of chronic pain, and pain thresholds using the pressure pain test in children with ADHD and healthy controls and examined the relation of MPH treatment on these parameters. The presence of chronic pain and pain perception were similar between the groups. Children with ADHD had lower pain thresholds than the healthy controls. However, lower regional pain thresholds were observed in the ADHD group without MPH compared to both the healthy control and ADHD with MPH groups. Although the pain thresholds of the ADHD with MPH group were regionally lower than those of the healthy controls, low pain thresholds were observed in fewer regions compared to the ADHD without MPH group.

To the best of our knowledge, this study is the first to evaluate both chronic pain perception and pain thresholds in children with ADHD using the pressure pain test applied to eight parts of the body. This study contributes to the literature by showing that pain thresholds are low in children with ADHD and supports previous research focusing on adults [12, 30]. Similarly to these studies, children with ADHD were more sensitive to pain sensation, and MPH may help normalize the pain experienced by these individuals by raising their pain thresholds. However, our findings were inconsistent with some previous studies assessing pain thresholds. Scherder et al. reported that children with ADHD exhibited lower pain perception than their healthy siblings. In that study, siblings were enrolled instead of healthy controls, and psychiatric disorders other than ADHD were not excluded in the siblings [28]. Northover et al. found that ADHD may be associated with high pain thresholds. However, that study compared pain sensitivity in children with ADHD with and without diagnoses of comorbid conduct disorder, and no healthy control group was enrolled. It was not therefore possible to determine whether individuals with ADHD have lower or higher pain sensitivity. However, the association between ADHD severity and higher pain sensitivity in that study is compatible with our own findings concerning the disorder [29]. However, the present study found no relation between symptom severity and pain thresholds. Studies with larger sample sizes are now needed to clarify this relationship.

Scarce and inconsistent findings have been reported regarding changes in pain perception in children with ADHD. Among the previous studies evaluating pain perception in ADHD, Scherder et al. found no difference in pain perception compared to controls, a finding consistent with the present study [28]. A different study evaluating the effect of MPH treatment on pain perception found that children and adolescents with ADHD without MPH treatment exhibited lower pain perception compared to both healthy controls and ADHD patients treated with MPH [39]. The difference may be attributable to methodological differences in the assessment of pain perception. Wolff et al. did not exclude additional psychiatric disorders in their study, and used parental ratings to assess pain perception. However, external ratings may not be accurate since pain is a subjective perception and cannot be accurately evaluated by someone other than the individual concerned (31). At the same time, in that study, children with ADHD were asked to estimate the perceived severity of pain deriving from painful events experienced in the previous three months. The present study evaluated the perceived severity of chronic pain (persisting for more than three months) experienced by children with ADHD without additional psychiatric disorders. As children with ADHD were still experiencing this pain, it may be assumed that less memory bias was involved in determining the perceived severity of pain. However, the suggestion in that study that MPH increases the perception of pain cannot be completely excluded in the present research, because we did not consider the duration of medication use in the children in the treatment group. The use of medication may have normalized pain perception in children with ADHD. A higher prevalence of chronic pain was observed in women diagnosed with autism spectrum disorder (ASD) and/or ADHD in one study [31]. The fact that only women were selected in that study and that diseases that may affect pain perception, such as ASD, were included may explain the discrepancy in the present study.

Deficits in time perception [40], emotional perception skills [41], and self-awareness of cognitive functions have been reported in children with ADHD [42, 43]. Boys with ADHD tend to exaggerate their performances in order to maintain a positive self-image [44]. Our study found a lower pain threshold in the ADHD group, which may have led children with the disorder to report more chronic pain perception. However, pain perception was similar between the groups in the present study. This may be attributable to the low awareness of sensory perception in children with ADHD. Another explanation may involve an effort to underestimate their pain, similar to the positive bias in children with ADHD concerning their competence.

The low pain threshold in ADHD may be co-present with the etiological consequences of the disorder. First, dysfunction of the locus coeruleus (LC) has been described in ADHD through a defect in the number of noradrenergic receptors [45]. A deficiency in the LC/noradrenergic system [46], part of the pain system [47] and which exhibits pain-suppressive properties, may play a role in the reduced pain threshold in ADHD [28]. Second, dopamine has been shown to affect the stages of pain processing [21]. It has been suggested that dysfunction of dopamine pathways in ADHD [48] may lead to changes in pain thresholds. Third, corticotropin-releasing hormone (CRH), secreted by the hypothalamic-pituitary-adrenal (HPA) system, exhibits an analgesic effect [49]. The decreased CRH activity due to the hypoactive HPA axis observed in ADHD [50] may lead to a lower pain threshold. Dysfunction in these three pain-related systems may cause children with ADHD to be more sensitive to painful stimuli by lowering the pain threshold. Studies have reported that individuals who have experienced significant pain in their lives have a lower tolerance to cold pain [51]. Individuals with ADHD are more likely to experience painful situations due to suffering more accidents and greater exposure to medical procedures [52]. The greater pain experiences in children with ADHD may increase pain sensitivity by lowering the pain threshold.

The model of the vigilance regulation in ADHD (hypoarousal hypothesis) interprets patients’ hyperactivity and sensory input seeking as an autoregulatory attempt to balance wakefulness by creating a stimulating environment. Hypoarousal in ADHD has mainly been demonstrated by means of skin conductance and electroencephalography [53]. The low pain threshold in ADHD may make these patients more sensitive to painful stimuli due to autoregulatory sensory seeking. Stimulants effectively reduce attention deficits and sensation-seeking behavior in individuals with ADHD [54, 55]. This may explain the change in pain threshold in our ADHD group treated with MPH.

In the present study, the pain thresholds of the ADHD with MPH group were higher than those of the group not receiving MPH. The analgesic effect of MPH treatment has been reported in several chronic pain conditions [56, 57]. In a recent randomized placebo-controlled trial, MPH was found to cause an increase in the cold pain threshold in healthy men [58]. Studies have shown that dopamine agonists such as amphetamine cause opioid secretion in healthy individuals [59, 60]. The release of opioids after stimulants in children with ADHD may explain the antinociceptive effect and the change in pain threshold in the treatment group. MPH can also inhibit norepinephrine reuptake in addition to the dopaminergic system [61]. The analgesic effects of drugs with norepinephrine reuptake inhibitor properties [62] may explain the difference in the pain threshold in the ADHD with MPH group. However, the higher pain threshold value in the ADHD group receiving MPH may also be due to the drug’s general therapeutic effect [12]. The increase in the ability to maintain performance of an activity and task with treatment in individuals with ADHD may explain the change in pain threshold.

The particular strengths of this study are that pain thresholds were measured using induced pain, and that the clinician who measured this was blinded to the study groups. Research has emphasized that MPH affects pain perception and thresholds in ADHD, and this effect should be evaluated [12, 29]. The fact that the treatment-naïve ADHD group in the present study had never used MPH before because they had not been previously diagnosed showed that the change in pain threshold in ADHD was independent of the drug’s therapeutic effect. Other strengths of this study are that it considered the patients’ medication use status and excluded those with additional psychiatric disorders, in contrast to other studies of pain in ADHD.

However, this study also has a number of limitations. Pain threshold and pain perception are distinct concepts. This study evaluated pain perception in chronic pain and pain thresholds with induced pain. Investigating pain perception during induced pain may lead to a better understanding of these pain-related variables. Using physiological measures (e.g., electrodermal activity) to assess sensitization to induced pain may yield a better understanding of pain perception changes in children with ADHD. Pain thresholds may vary due to exposure to external events, such as harsh parental discipline and peer bullying [29]. Experiencing the pain of social rejection, exclusion, or loss involves the same neural areas that process physical pain [63]. The relationship between pain perception and ADHD should be evaluated by considering gender and the type of ADHD, and also comorbid psychiatric conditions. Future research should be designed to take these factors into account.

## Conclusions

The findings of this study show that children with ADHD have a low pain threshold, and that MPH may raise this. Experimental investigation of the dopaminergic and opioid systems in the brain will provide a better understanding of pain changes in ADHD. Families and clinicians must be aware of situations that may cause pain in children with ADHD. These patients’ low threshold for pain may lead them to experience more pain. Finally, the fact that children with ADHD do not report pain to painful stimuli does not necessarily mean that they do not experience it.

## Data Availability

Applicable.
